# Comparing the Structure of Microgels at Liquid–Liquid and Solid–Liquid Interfaces

**DOI:** 10.1021/acs.langmuir.5c02599

**Published:** 2025-06-20

**Authors:** Rodrigo Rivas-Barbosa, Fabrizio Camerin, Jacopo Vialetto, Shivaprakash N. Ramakrishna, Lucio Isa, Emanuela Zaccarelli

**Affiliations:** † School of Physics and Astronomy, University of Edinburgh, Edinburgh EH9 3FD, U.K.; ‡ Division of Physical Chemistry, Department of Chemistry, Lund University, P.O. Box 124, Lund SE-22100, Sweden; § Department of Chemistry, 9300 University of Florence, via della Lastruccia 3, Sesto Fiorentino, Firenze I-50019, Italy; ∥ Consorzio interuniversitario per lo sviluppo dei Sistemi a Grande Interfase (CSGI), via della Lastruccia 3, Sesto Fiorentino, Firenze 50019, Italy; ⊥ Laboratory for Soft Materials and Interfaces, Department of Materials, ETH Zürich, Vladimir-Prelog-Weg 5, Zürich 8093, Switzerland; # CNR Institute of Complex Systems, Uos Sapienza, Piazzale Aldo Moro 2, Roma 00185, Italy; ¶ Department of Physics, Sapienza University of Rome, Piazzale Aldo Moro 2, Rome 00185, Italy

## Abstract

The adsorption of soft microgels at interfaces is a key phenomenon in numerous applications, including emulsification, interfacial stabilization, and responsive coatings. In this work, we employ a combination of in situ atomic force microscopy and numerical simulations to systematically investigate the structural behavior of individual microgels at liquid–liquid and solid–liquid interfaces. By directly comparing their morphology and response to temperature variations, we examine the changes in their overall conformation and the evolution of their internal polymer distribution, uncovering key differences between the two environments. Our results provide detailed insights into the microscopic changes occurring during the adsorption of soft, deformable particles at interfaces, highlighting both new pathways for controlling their interfacial properties and the necessity of advanced characterization techniques to fully capture these complex processes.

## Graphical abstract

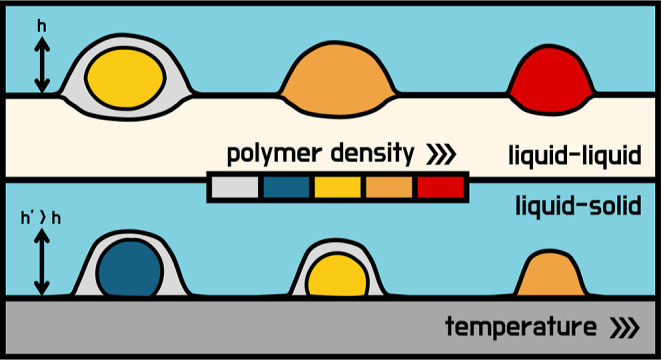

## Introduction

Microgels at liquid–liquid interfaces exhibit unique behavior due to their dual polymeric-colloidal nature. Specifically, they undergo deformation and spreading within the interface, effectively reducing the surface tension between the two immiscible fluids. The extent of deformation at the interface is dictated by the microgel size, softness, and internal architecture,^1,2^ as well as by their solubility in the liquid phases.^3^ In particular, for standard core–corona microgels synthesized via radical precipitation polymerization, their structure below the volume phase transition temperature (VPTT) is that of a fried egg,^3–9^ thus deviating from the standard spherical shape observed in bulk. This conformation arises due to the rigidity and higher cross-linking density of the core compared to the more flexible, less cross-linked shell.^10^

Due to their deformability, extensive spreading at interfaces, and partial responsiveness maintained by the polymer, microgels adsorbed at interfaces find applications across a wide range of fields. To name a few examples, they can be used as temperature-sensitive emulsifiers,^11–13^ as emulsion chambers for enzyme biocatalysis,^14^ to fabricate lithography masks for nanowire arrays with controlled spacing, width, and height,^15–17^ and for creating hierarchical complex patterns by consecutive deposition of multiple layers on a solid surface.^18^

On a fundamental level, the characterization of the microgel morphology and the evaluation of their responsiveness to changes in temperature at the interface mainly rely on experiments conducted on solid substrates after deposition from fluid interfaces. In these conditions, for standard core–corona microgels, it is normally assumed that the in-plane lateral extension of the microgels remains unchanged when transferred onto the plate.^5,15,16,18–26^ In fact, most of the work in this regard has used atomic force microscopy (AFM) as a tool to characterize the microgel profiles and the structures obtained upon varying interfacial compression before deposition. In this respect, important remarks must be made. For instance, at the single-particle level, it was explicitly shown that this technique does not allow for a reliable measure of the size when particles with a rigid core and soft-shell architectures are used. In this case, the extent of the error in the measured size of the deposited particles increases for thin shells due to the hard core hampering polymer deposition on the substrate.^27^ Other studies have focused on understanding the effect of surface coating and deposition procedure on the microgel morphology after adsorption.^28,29^ By reconstructing the shape of the microgel with super-resolution fluorescence microscopy,^30,31^ it was demonstrated that, although the deposition method initially determines the microgel deformation at the interface, after rehydration, the shape is essentially maintained, at least in the case of standard microgels. The case of ultralow cross-linked microgels instead presents marked differences, where the strong deformation that is seen in the preparation of the sample by either spin-coating or by Langmuir–Blodgett deposition can only be avoided via in situ adsorption onto the substrate.^32^ Additional care must be taken with low cross-linked microgels, as it has been shown that elevated temperatures can form persistent intra- and interpolymer chain hydrogen bonds. These bonds effectively act as additional cross-linkers causing hysteresis during swelling/deswelling cycles.^33^

More recent studies have also focused on the collective response of a microgel monolayer on different substrates, showing that the deposition protocol can alter the structural properties of the colloidal monolayer.^34^ In addition, soft microgels and hydrophobic substrates affect the particle morphology less than harder microgels and hydrophilic substrates.^35^ Moreover, the long-debated presence of a structural transition observed for these particles^36–38^ may depend on the deposition and subsequent drying process.^39^ Ultimately, these works indicate that ex situ measurements on solid substrates have to be taken with caution, because of important differences with in situ approaches that may reflect on the single-particle structure.

Advancing toward a more accurate and representative understanding of the conformation of soft colloids, recent developments in experimental techniques have led to the extension of the in situ AFM originally devised for solid–fluid interfaces^40–42^ to fluid–fluid ones.^8,43,44^ This extended method represents a significant step forward in enabling the 3D characterization of microgels in their native environment by capturing their structure on both sides of the interface through combined imaging from the two fluid phases. This technique was successfully used for both standard and core–shell microgels. Similarly, another valuable alternative are numerical simulations. In this context, explicit solvent simulations have been extensively employed in the recent past to study microgels at the liquid–liquid interface with standard^5,6,19,26,45^ and more complex architectures.^23,43,46^

In this work, also motivated by the recent results, we investigate the structure of individual microgels at solid–liquid and liquid–liquid interfaces and analyze how these structures change with increasing temperature. This allows us to carefully address similarities and differences between the arrangements under the two different conditions investigated. To this aim, we first characterize the conformation and temperature response of the microgels directly at a liquid–liquid and a solid–liquid interface by employing in situ AFM.^8^ For both interfaces, we observe a consistent deswelling with increasing temperature of the part of the microgel exposed to water, with a reduction of both height and width due to the collapse of the polymer network above the VPTT. On the contrary, on the oil side of the liquid–liquid interface, we detect a subtle increase in the height profile with rising temperature. These observations are consistent with results from computer simulations, which we further leverage to investigate the internal material distribution and gain insights into the resulting profiles. Additionally, at the solid–liquid interface, the density profiles indicate the formation of layers composed of polymer chains aligned parallel to the interface at higher temperatures, a feature that is not present at a liquid–liquid interface. Finally, simulations also reveal which microgel polymers are adsorbed at the interface, highlighting significant differences between the two investigated conditions. At the solid–liquid interface, adsorption occurs almost entirely via the microgel corona, whereas at fluid interfaces, it also involves polymers belonging to the inner core.

## Methods

### Numerical Details

#### Microgel Modeling

Microgel particles with a disordered topology are assembled following the procedure described in ref 47. Microgels realized with this method share the same structural properties as those synthesized in the lab, that is, a predominantly spherical shape in bulk with a compact core and a loose corona. The form factor and density profile have been corroborated through comparison with X-ray scattering and super-resolution microscopy experiments.^31,47,48^ The microgel assembly process was sped up by performing it on GPUs using the oxDNA package.^49^

Once assembled, the interaction between beads (also referred to as monomers) composing the microgel follow the bead–spring Kremer–Grest model.^50,51^ The overlapping of monomers is inhibited by the Weeks–Chandler–Andersen (WCA) potential^52^

1 $$V_{WCA}\left(r\right)=\{\begin{array}{ll} 4\varepsilon \left[{\left(\frac{\sigma }{r}\right)}^{12}-{\left(\frac{\sigma }{r}\right)}^{6}\right]+\varepsilon & r\leq 2^{1/6}\sigma \\ 0 & r>2^{1/6}\sigma , \end{array}$$

with σ being the diameter of each bead, and the unit of length in simulations, and ε setting the energy scale. The topology of the network is kept fixed with permanent bonds between monomers. This is achieved by having the connected monomers additionally interacting via the finite extensible nonlinear elastic potential (FENE)^53^ defined by eq 2.

2 $$V_{FENE}\left(r\right)=-\varepsilon k_{F}R_{0}^{2}\ln\left[1-{\left(\frac{r}{R_{0}\sigma }\right)}^{2}\right],r<R_{0}\sigma$$

The dimensionless spring constant and the maximum extension of the bond have values of *k*_F_ = 15 and *R*_0_ = 1.5, respectively.

For some of the simulated scenarios, the temperature-responsive behavior of the microgels is emulated with the use of a solvophobic (attractive) interaction $V_{\alpha_{mm}}\left(r\right)$ acting among monomers,^54,55^ which reads

3 $$V_{\alpha_{mm}}\left(r\right)=\{\begin{array}{ll} -\varepsilon \alpha_{mm} & r\leq 2^{1/6}\sigma \\ \frac{1}{2}\varepsilon \alpha_{mm}\left[\cos\left(\gamma {\left(\frac{r}{\sigma }\right)}^{2}+\beta \right)-1\right] & 2^{1/6}\sigma <r\leq R_{0}\sigma \\ 0 & r>R_{0}\sigma , \end{array}$$

with the α_mm_ parameter encoding the solvent conditions. An increase in the strength of α_mm_ leads to a rise in the monomer–monomer attraction, causing the microgel to shrink, hence replicating the worsening of the solvent conditions. Here, $\gamma =\pi {\left(2.25-2^{1/3}\right)}^{-1}$ and β = 2π – 2.25γ fulfill the potential and its derivative to be continuous at *r* = 2^1/6^σ and *r* = 1.5σ.

#### Explicit Solvent Modeling

Simulations with explicit solvent are carried out using dissipative particle dynamics (DPD),^56–60^ a technique that groups solvent molecules into coarse-grained beads. The total force acting on a pair of solvent beads can be split into three terms: a conservative (eq 4), a dissipative (eq 5), and a random force (eq 6), so **F**_*ij*_ = **F**_*ij*_^C^ + **F**_*ij*_^D^ + **F**_*ij*_^R^ given by

4 $$F_{ij}^{C}=\{\begin{array}{ll} a_{ij}\left(1-r_{ij}/r_{c}\right){\mathrm{r̂}}_{ij} & r_{ij}<r_{c}\\ 0 & otherwise. \end{array}$$

5 $$F_{ij}^{D}=-\gamma w^{2}\left(r_{ij}\right)\left(v_{ij}\cdot {\mathrm{r̂}}_{ij}\right){\mathrm{r̂}}_{ij}$$

6 $$F_{ij}^{R}=\sqrt{2\gamma k_{B}T}w\left(r_{ij}\right)\frac{\zeta_{ij}}{\Delta t}{\mathrm{r̂}}_{ij}$$

with the species-dependent *a*_*ij*_ controlling the maximum repulsion between *i* and *j* beads (commonly referred to as the DPD parameter), *r*_*c*_ being the cutoff radius, **r**_*ij*_ = **r**_*i*_ – **r**_*j*_, *r*_*ij*_ = |**r**_*ij*_|, ${\mathrm{r̂}}_{ij}=r_{ij}/r_{ij}$ , the weight functions given by *w*(*r*_*ij*_) = 1 – *r*_*ij*_/*r*_*c*_ for *r*_*ij*_ < *r*_*c*_ and 0 otherwise, γ being a friction coefficient, and ζ_*ij*_ being a random number with zero mean and unit variance. The value of these parameters and the reduced solvent density ρ̅ are included in Table 1.

**Table 1:** System Details and Integration Schemes for the Modeled Systems

System	liquid–liquid modified DPD (A)	liquid–liquid traditional DPD (B)	solid–liquid
simulation technique	DPD		Langevin
	*r*_*c*_ = 1.9σ, γ = 2.1, *a*_ww_ = *a*_hh_ = 8.8, *a*_wh_ = 31.1, ρ̅ = 4.5		$damp=200\times \sqrt{m\sigma^{2}/\varepsilon }$
$V_{\alpha_{mm}}$ potential	yes	no	yes
T* = *k*_B_ *T*/ε	1		
microgel monomers	41182, 5% cross-linkers		
solvent particles	2015454		
surface particles			115200
box size [σ^3^]	160 × 160 × 120		240 × 240 × 120
time step $\left[\times \sqrt{m\sigma^{2}/\varepsilon }\right]$	0.002		
steps	>7 × 10^6^		2 × 10^7^

#### Solid Surface Modeling

Following the methodology presented in ref 31, a monomer-impermeable solid surface is modeled with a wall composed by two layers of immobilized surface particles. The second layer, placed 0.6σ below the first one, is used to avoid microgels penetrating into the wall. For each layer, surface particles of diameter *d* = σ are initially placed in a square lattice of side size σ; the positions are then slightly shifted by randomly displacing them in all directions following a Gaussian distribution with mean μ = 0 and standard deviation σ_sd_ = 0.2 to avoid crystallization of the microgel beads. The obtained layers are subsequently kept immobile throughout the entire simulation runs. The interaction between microgel monomers and surface particles is given by the sum of the repulsive *V*_WCA_ and the solvophobic $V_{\alpha_{ms}}$ potentials (eqs 1 and 3). The “ms” subscript is used to distinguish the solvophobic monomer–surface from the monomer–monomer interaction parameters. The strength of the monomer–surface attraction is chosen according to the nature of the surface. For instance, for a hydrophilic wall, the monomer–surface interaction should be purely repulsive, so α_ms_ = 0. Instead, for the opposite case of a hydrophobic wall, α_ms_ > 0 captures the affinity of the microgel to the surface; in particular, based on our previous study,^31^ the results presented here are for a monomer–surface attraction of α_ms_ = 1.0. In contrast to the previous work, the microgel is secured next to the surface by adding a gravity-like force acting on the monomers. The inclusion of gravity is particularly important for the case of a hydrophilic wall, since otherwise the microgel would drift away from the surface. Here, a sedimentation-like process is modeled, with the microgel initially immersed in the liquid phase and then settling at the solid–liquid interface. It is worth noting that, in contrast to the experiments, the initial state here is not a deformed microgel adsorbed at the liquid–liquid interface. The strength of the gravity force is set to F_*g*_ = 0.0007 [ε/σ], as this choice proves sufficient to keep the microgels next to a hydrophilic wall, while retaining a density profile similar to when in bulk, in accordance to previous super-resolution microscopy measurements^31^ (see Supporting Information Section S1).

#### Simulation Parameters

Depending on the system modeled, the simulation technique and interactions are chosen to use the computational resources efficiently. In particular, results for the liquid–liquid interface scenario were obtained with a mixed DPD simulation technique (see also below). For the solid–liquid interface, Langevin simulations are performed with the solvent modeled implicitly. Table 1 summarizes the diverse combination of techniques, interactions, and size of the systems employed. All simulations were performed using LAMMPS.^61^

#### Calculated Quantities

The microgels at the different interfaces are characterized by density and height profiles. In particular, the microgel density parallel to the interface ρ­(*z*) (liquid–liquid or solid–liquid) is calculated by slabbing the box along the *z* axis, with the plane of the interface at *z* = 0, and counting the amount of beads in each bin. Similarly, ρ­(ζ) represents the density perpendicular to the interfacial plane, with ζ = *x*, *y*, and it is averaged over both directions; the profile is aligned such that the *x* and *y* components of the center of mass (CM) are located at ζ = 0. In addition, with the aim to have a quantity closer to the AFM measurements, numerical height profiles *h*_*z*_(ζ) are calculated from the position of the beads that protrude the most on each side of the interface. To obtain these profiles, first, a thin slice of the system of width σ is taken perpendicularly to the interfacial plane and passing through the microgels *xy* center of mass. Next, this slice is slabbed along the long axis, similar to ρ­(ζ). Finally, for each slab in the slice, the height of the bead furthest from the interface is recorded. The height profile for a given configuration is thus the average of the recorded values obtained by repeating the procedure 20 times for slices at different cutting angles. By slabbing the slice a second time, now parallel to the interface, a grid is obtained from which the slice density can be calculated, providing information about the inner structure of the microgel beneath the numerical height profiles. These slice densities are presented in color maps and are calculated at the microgels *x* and *y* components CM. For all densities and height profiles, the reported values are the average over more than 200 configurations taken in different time steps.

#### Validation of the Modified DPD Method

In a previous study by some of us,^7^ polymer profiles from simulations using a *modified* DPD approach were shown to be consistent with neutron reflectometry measurements. The modification consisted of the inclusion of an additional interaction between beads to model the effect of temperature (eq 3). In that approach, the monomer–solvent and solvent–solvent interactions are kept fixed, and it is only the rise of attraction parameter α_mm_ that drives the collapse of the microgel, modeling an increase in temperature. Despite the ability of the method to capture the temperature response successfully, here we assess its validity once again, this time by comparing its results with those from a more traditional approach, without additional potentials, where the shrinkage due to temperature effects is encoded in the monomer–solvent repulsion parameters (*a*_mx_ from eq 4). To enhance the readability, the modified and traditional DPD models are denoted as A and B, respectively.

The validation of the approach is condensed in Figure 1, with the explanation of each panel given hereafter. Method A is validated by demonstrating that the structure adopted by microgels, above, below, and near the VPT using this approach, can also be obtained via the traditional B method, therefore establishing a relation between the α parameter of A with the *a*_mx_ parameters of B. From a fundamental perspective, the nature of how these two methods model temperature is different. On the one hand, the A approach involves a pull interaction between microgel beads to mimic changes in temperature. On the other hand, in method B, i.e., the traditional approach, it is the change in the monomer–solvent push interaction that models variations in temperature. The difference between the A and B methods is illustrated in the first column of Figure 1a. The top figure, corresponding to method A, in addition to the blue and yellow arrows displaying the force exerted onto the microgel from the water and oil beads (respectively, eq 4), includes red arrows representing the attractive nature from the monomer–monomer *V*_α_ interaction (eq 3). In contrast, the bottom figure, representing method B, lacks the red arrows but displays thicker blue and yellow arrows, indicating a stronger repulsion from the solvent to the microgel compared to that of method A.

**Figure 1:**
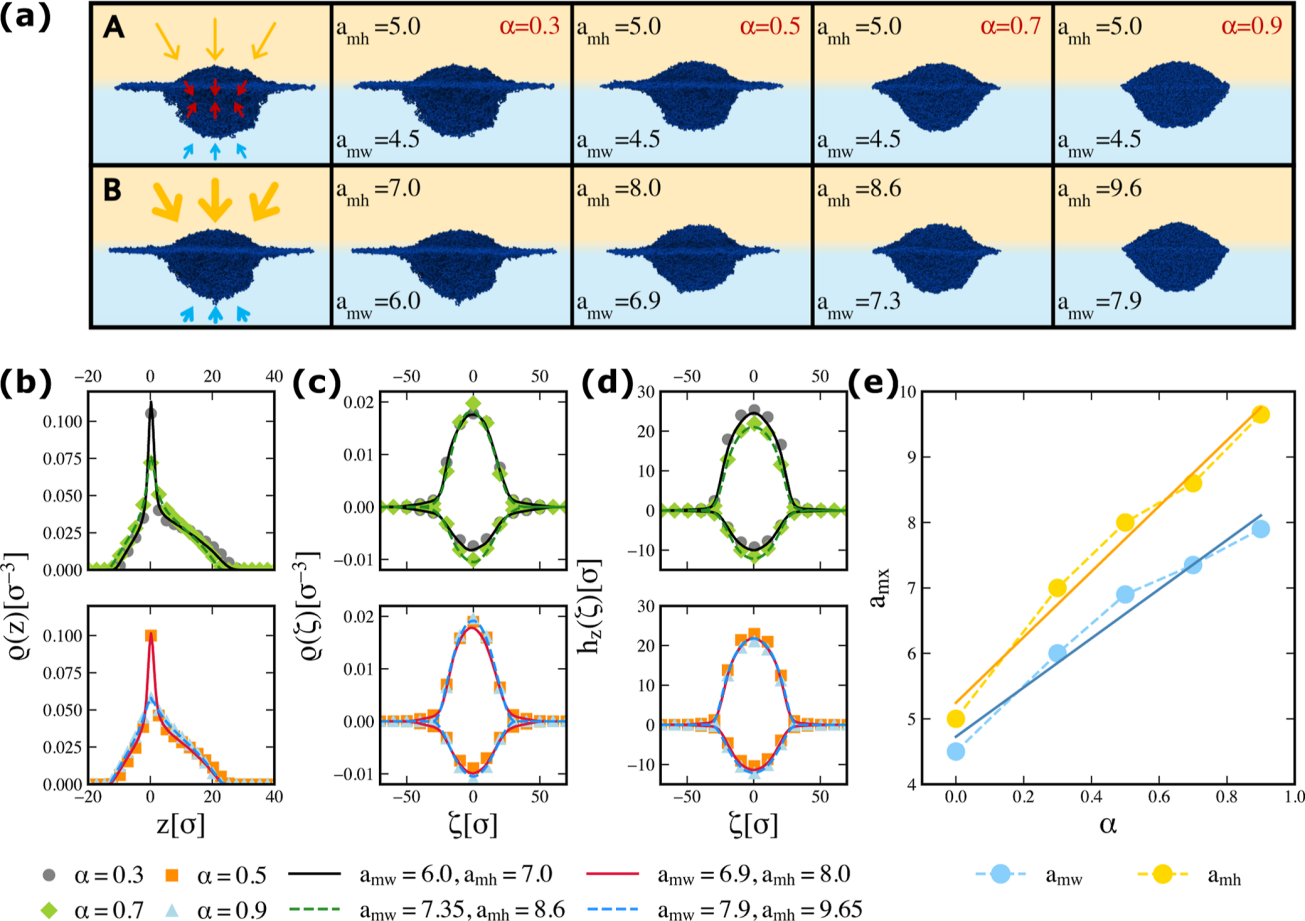
(a) Representative side-view snapshots of a N ≈ 42k microgel with *c* = 5% at different effective temperatures α and for different values of the *a* interaction parameter. The top (bottom) panels correspond to the A (B) approach. (b,c,d) Density profiles as a function of the effective temperature showing ρ­(*z*), ρ­(ζ) and *h*_*z*_(ζ), respectively. Profiles obtained with the A (B) simulation approach are shown with symbols (lines). The water (oil) side is on the positive (negative) values of the *x*-axis for panel (b) and *y*-axis for (c,d) to allow for an easier comparison later on with the profiles calculated at the solid–liquid interface. (e) Interaction parameters *a*_mx_ as a function of α. Solid lines are linear fits.

Although the interaction mechanisms differ between the two methods, their equivalence can be established if the microgels adopt the same structure in both cases; that is, when the resulting density profiles of the microgels are identical. To this aim, the density profile of microgels parallel to the interface, ρ­(*z*), simulated using method A at four different effective temperatures (α = 0.3, 0.5, 0.7, and 0.9) are matched with those of simulations performed with the method B by varying the *a*_mx_ parameters until an equivalent profile is found. Representative snapshots of the simulations with the A approach and their equivalent B parameters are shown in Figure 1a for the four different tested effective temperatures, with the corresponding parameters labeling the snapshot. The parallel and perpendicular density profiles, as well as the height profiles, are displayed in Figure 1b–d, showing good agreement between the approaches. To make the comparison of the water side of the profiles with those from the solid–liquid case easier, the water side is set on the positive values of the *x*-axis for panel (b), and on the positive values of the *y*-axis for panels (c) and (d). Finally, in panel (e), the matching *a*_mx_ parameters are reported as a function of α. Both values of *a*_mx_ increase with α, as expected, since the quality of the solvents worsens by raising temperature. Their relative dependence is well captured by a linear fit, suggesting that the *a*_mx_ parameters linearly increase with α but at different rates relative to each other. In particular, the monomer–oil parameter is found to grow faster than the monomer-water one.

Overall, the good agreement of the structural properties of the microgels simulated with both methods validates the mapping between them. For any simulation with the mixed A approach, there is an equivalent set of B parameters. Additionally, there is an apparently linear scaling of the *a*_mx_ parameters with α. This linearity can be exploited to estimate the temperature mapping between both methods. At last, it is worth mentioning that the computational times are very similar for the two methods: for A, which has an additional interaction, simulations are only roughly 1% slower than for the B approach. More details on the validation procedure, including the original mapping found using a scaled system (Section S2), the linearity of the *a*_mx_ parameters as a function of α (Section S3), and its applicability to microgels with a different cross-linker content (Section S4), are discussed in the Supporting Information.

## Experimental Details

### Reagents

*N*,*N*′-Methylenebis­(acrylamide) (BIS, Fluka, 99.0%), methacrylic acid (MAA, Acros Organics, 99.5%), potassium persulfate (KPS, Sigma-Aldrich, 99.0%), isopropanol (Fisher Chemical, 99.97%), toluene (Fluka Analytical, 99.7%), *n*-hexane (SigmaAldrich, HPLC grade 95%), and *n*-hexadecane (Acros Organics 99.0%) were used without further purification. *N*-isopropylacrylamide (NIPAM, TCI 98.0%) was purified by recrystallization in 40/60 v/v toluene/hexane. Water used for all experiments is bidistilled Milli-Q, with a pH of ≃ 6.

### Microgel Synthesis

The synthesis of the microgels used in this study is already reported elsewhere.^8,62^ Briefly, the microgels are composed of NIPAM (1 g) as the main monomer, with the addition of 5 mol % MAA and 5 mol % BIS. For their synthesis, we used a semibatch protocol. Monomers and cross-linkers dissolved in 50 mL of Milli-Q water were purged with nitrogen for 1 h. Then 4/5 of the reaction mixture was transferred to a syringe, and 10 mL of Milli-Q water was added to the reaction flask. The solution was then immersed in an oil bath at 80 °C and purged with nitrogen for another 30 min, and the reaction was initiated by adding 13 mg of KPS previously dissolved in 1 mL of Milli-Q water and purged with nitrogen. After 1.5 min, we started feeding of the separate monomers solution at 0.5 mL/min. After injecting all of the solution, the reaction was quenched by opening the flask to oxygen and placing it in an ice bath. The obtained colloidal suspension was cleaned by dialysis for a week and by 8 centrifugation cycles and resuspension of the sedimented particles in pure water.

### Dynamic Light Scattering

Dynamic light scattering (DLS) experiments were performed on a Zetasizer instrument (Malvern, UK). The microgels were dispersed in Milli-Q water at a concentration of 0.01 wt %. The temperature was scanned from 20 to 50 °C in 2 °C steps. Each temperature was allowed to equilibrate for 10 min before four consecutive measurements of 15 runs each were performed.

### AFM at the Liquid–Liquid Interface

A detailed description of the protocol used for AFM imaging at the liquid–liquid interface is reported in refs 8 and 43 . Here, we briefly recapitulate the main piece of information. We used an AFM (Dimension Icon, Bruker) with PeakForce Tapping mode and cantilevers with a nominal spring constant of ∼0.12 N·m^–1^ (PEAKFORCE-HIRS-F-B, Bruker). The sample cell has a reservoir with a depth of around 10 μm containing the subphase made of UV curable resin on a silicon substrate. For imaging from the oil phase, the reservoir was filled with approximately 5 μL of a dilute microgel suspension in water. Afterward, the entire cell was filled with hexadecane. For imaging from the water side, the reservoir was first filled with hexadecane, then the fluid surface covered with ∼5 μL of the microgel suspension in water. After 5 min, the entire cell was flushed with water to remove any excess of microgels floating in the bulk phase. The sample cell was glued to a temperature-controlled cell (MFP 3D, Asylum research, Oxford instrument), and the temperature was set to the required value. Imaging started around 30 min after the temperature-controlled cell was placed under the AFM. This waiting time allows for the cell temperature to equilibrate. After this equilibration time, drift at the interface was greatly reduced, allowing for the stable capture of high-resolution images. For imaging, the tip was approached to the interface by setting a PeakForce set point of approximately 100 pN. To optimize the image quality, the set point was slightly adjusted along with the feedback gains once the tip was engaged at the interface. The PeakForce during the imaging was varied between 100 pN and 500 pN. The PeakForce amplitude during imaging in the various fluid phases was varied between 100 and 300 nm. The oscillation frequency was chosen between 1 and 2 kHz, and the scanning rate was chosen between 0.2 and 1 Hz.

### Microgels Deposition on the Solid Substrate

Microgels were deposited on a silicon wafer from a hexane–water interface following an already reported procedure.^23^ Silicon wafers were first cleaned by 15 min ultrasonication in the following solvents: toluene, isopropanol, acetone, ethanol, and Milli-Q water; following by 10 min in a UV-Ozone cleaner (UV/Ozone Procleaner Plus, Bioforce Nanosciences) just before starting the experiments. This ensured a highly hydrophilic surface. One silicon wafer was placed inside a Teflon beaker on the arm of a linear motion drive and immersed in water. Successively, a liquid interface was created between water and *n*-hexane. Around 100 μL of the microgels suspension was injected at the interface after appropriate dilution in a 4:1 Milli-Q-water/isopropanol suspension. After 10 min equilibration time, extraction of the substrate was conducted at a speed of 25 μm s^–1^ to collect the microgels adsorbed at the liquid interface and transfer them on the silicon wafer. The silicon wafers with deposited microgels were then reimmersed in Milli-Q water for AFM imaging.

### AFM at the Solid–Liquid Interface

Imaging the microgels on the solid substrates was also conducted using an AFM (Dimension Icon, Bruker) employing the PeakForce Tapping mode. For these measurements, cantilevers with a nominal spring constant of 0.35 N·m^–1^ (SNL-10, Bruker) were utilized. The substrate containing the microgel was positioned within a fluid cell and submerged in a Milli-Q water. Following a waiting period of approximately 15 min to allow for system equilibration, the cantilever was carefully brought into contact with the substrate. The PeakForce set point was then adjusted to ensure the acquisition of high-quality images, typically ranging between 1 and 2 nN. Throughout imaging at different temperatures, the PeakForce amplitude varied between 100 and 300 nm. Additionally, the oscillation frequency for PeakForce Tapping was set between 1 and 2 kHz, and the scan rate ranging from 0.2 to 1 Hz.

### AFM Image Analysis

All AFM images were analyzed with the open source Gwyddion software; basic image processing was made if necessary (with appropriate masks to account for the particle features) until the interface appeared leveled. Further analysis to extract the radial height profiles of the particles was performed by using custom-made MATLAB codes.

## Results and Discussion

We begin by describing the configuration of the microgels as captured from their height profiles, *h*_*z*_(ζ), with ζ representing the axes parallel to the interfacial plane, as sketched in Figure 2. To facilitate the comparison of the profiles between interfaces, we set the *z* axis positive on the water side. Having settled the geometry and frame of reference of our systems, we now pass to describe the experimental height profiles *h*_*z*_(ζ) obtained from in situ AFM. These are shown at the solid–liquid (silicon wafer-water) and liquid–liquid (hexadecane-water) interface in Figure 3a for three different temperatures, increasing from left to right, comprising the VPTT at ≈32 °C (see Section S5). Focusing first on the data for microgels deposited onto a solid surface, displayed in solid lines, we observe that the maximum height of the peak centered at ζ = 0 decreases with temperature, pointing to the hallmark temperature response of pNIPAM-based microgels. The decrease in size is noticeable especially when moving from 25 to 35 °C, which indeed crosses the VPTT. We also observe that the in-plane diameter of the microgels, defined here as the width of the profile at the base of the curve, decreases with temperature. The in-plane diameter at 35 and 40 °C is approximately 75% and 70% of that at 25 °C, respectively.

**Figure 2:**
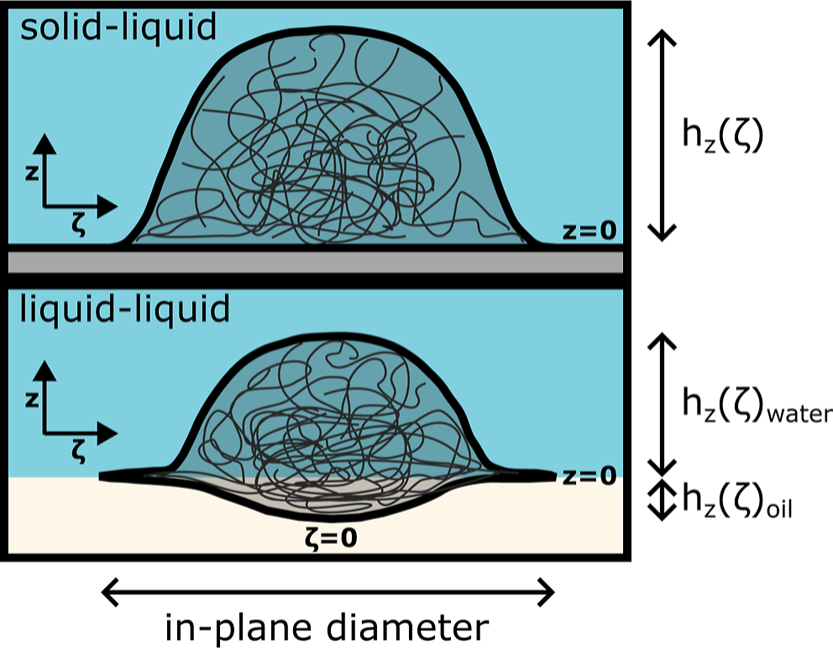
Sketch of a microgel at the solid–liquid and liquid–liquid interfaces. Positive *z* valus are for the water side.

**Figure 3:**
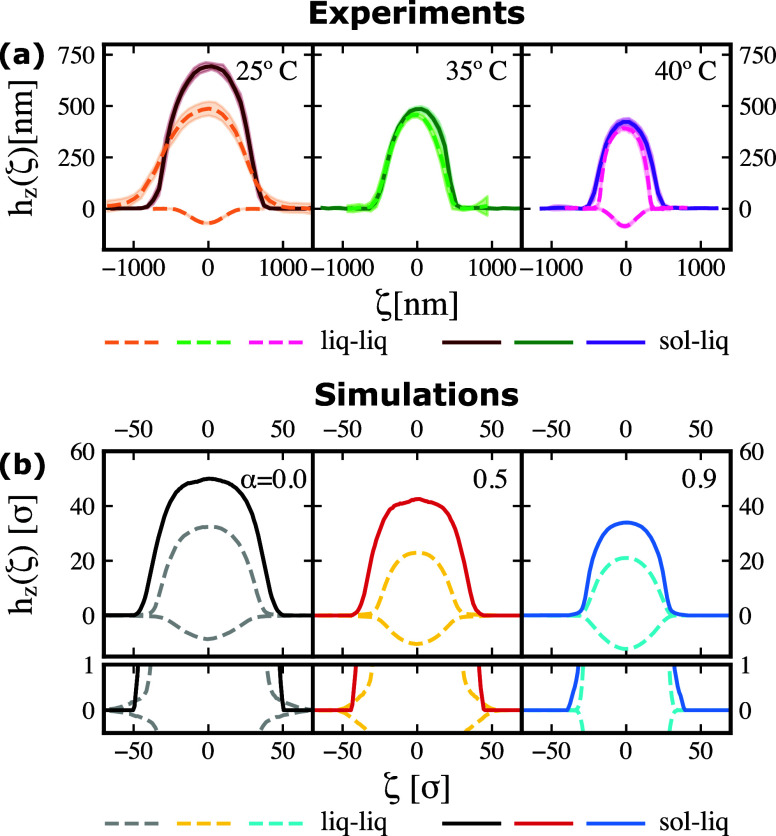
(a) Experimental and (b) numerical height profiles *h*_*z*_(ζ), with ζ being the axes parallel to the interfacial plane, of the microgel at liquid–liquid (dashed lines) and solid–liquid (full lines) interfaces. The temperature *T* and the effective temperature α increases from left to right. In (a), the shaded regions alongside the experimental curves are their corresponding standard deviation. In (b), the small lower panels are enlargements for the region close to the interface. For both panels, the water side is on the positive and the oil in the negative values of the *x*-axis. Experimental data for the oil side at intermediate temperature are not available.

Turning to the profiles of microgels adsorbed at the water–oil interface (dashed lines), a similar behavior is observed on the water side, where the height of the peak decreases with temperature. Likewise, the in-plane diameter shrinks with the temperature. This behavior is at odds with a previous study^19^ that noted that the lateral extension is largely temperature-independent. This discrepancy is probably due to the difficulty to detect sparse peripheral polymer chains at the interface, perhaps partly due to the lower resolution compared to the *ex situ* setup used in that study, but possibly also due to the polymer chains remaining mobile throughout the in situ scanning process. Contrary to the trend on the water side, the height of the peak on the oil side (negative values) increases with temperature, although in a more subtle manner and again accompanied by a decrease of the in-plane diameter. We speculate that at high temperatures, polymer chains from the water side get pushed toward the center of the microgel, located near the interface, supplying extra material to the oil side. This hypothesis will be verified later by simulations. These measurements also indicate that the in-plane diameter is influenced not only by temperature, as mentioned before, but also by the type of interface onto which microgels are adsorbed. At 25 °C, the in-plane extension is larger on the water side for the microgels at the liquid–liquid interface; however, the opposite occurs at 40 °C, where the in-plane diameter is slightly wider for the microgels deposited on the solid substrate. Again, we stress that the exact extent of deformation of the microgel on the interfacial plane, for both interfaces, suffers from resolution limitations due to the difficulty in detecting individual spread polymer chains at the particle periphery. Finally, we notice that the peak height and overall shape of the profiles on the water side at either interface look more alike above the VPTT, when the majority of the polymer network in water is collapsed.

We now proceed to discuss Figure 3b, which displays the height profiles calculated from the simulations. We recall that the effect of temperature is mimicked by using the solvophobic potential described in eq 3 and modulated by the α parameter. With this, bad solvent conditions are modeled with a growing value for such a parameter. This applies both to simulations at the liquid–liquid interface that are run in the presence of explicit solvent and to those at the solid–liquid interface where solvent is modeled implicitly. More details on the simulations are given in the Methods. We observe that the features of the numerical height profiles *h*_*z*_(ζ) are qualitatively similar to those obtained from experiments. Specifically, the height on the water side decreases with temperature for both interfaces, while the peak on the oil side increases. Additionally, the in-plane diameter shrinks irrespective of the solvent side or interface. Overall, the profiles on the water side for both interfaces get closer to each other with an increasing temperature. Another feature that is well captured by simulations is the crossover of the in-plane diameter with temperature: starting at low effective temperature α = 0.0, the liquid–liquid lateral extension is greater than the liquid–solid one (see lower panels zooming on the interfacial plane); however, at high effective temperature, that is at α = 0.9, the profile at the solid–liquid interface becomes the largest of the two.

Having discussed the overall conformation of the microgel, we now inspect its inner structure, thanks to the insights provided by the simulations. To begin with, we inspect the polymer density inside the microgel at α = 0.0; see Methods. This is shown in the top panels of Figure 4 together with the height profile curves shown as pink dashed lines. Starting with the solid–liquid case, we observe a nearly circular region of rather large density at the center, which then radially decays, as is expected from the characteristic core–corona architecture of these microgels. The radially decaying density gets disrupted at the interface (*z* = 0), where a thin layer with the largest density is detected, arising from polymer–substrate interactions. Similarly, in the liquid–liquid scenario, the microgel presents a radially decaying density but this time sharply disrupted not by a straight solid interface but convexly by the insolubility of the microgel network in oil. Notably, the density is mostly homogeneous on the oil side, where the polymer chains are collapsed, and well delimited by the height profile curve. Instead, on the water side, the density near the height profile curves smoothly changes along the contour, being relatively low near the edges, with respect to the center of the profile. The explanation behind the different density behaviors near the profile curves comes from the lack of hydration of the peripheral polymer chains. In fact, while on the water side, at this temperature, polymer chains are free to extend and diffuse around, on the oil side where interactions are less favorable, they are repelled more strongly by the solvent and pushed toward the interface. In fact, as shown in the lower panels of Figure 4, which report the density color-maps at higher temperatures, the radial difference in density for the water side narrows and gets more and more confined by the height profile curve. This is expected since the polymer beads experience an overall lower solvent quality.

**Figure 4:**
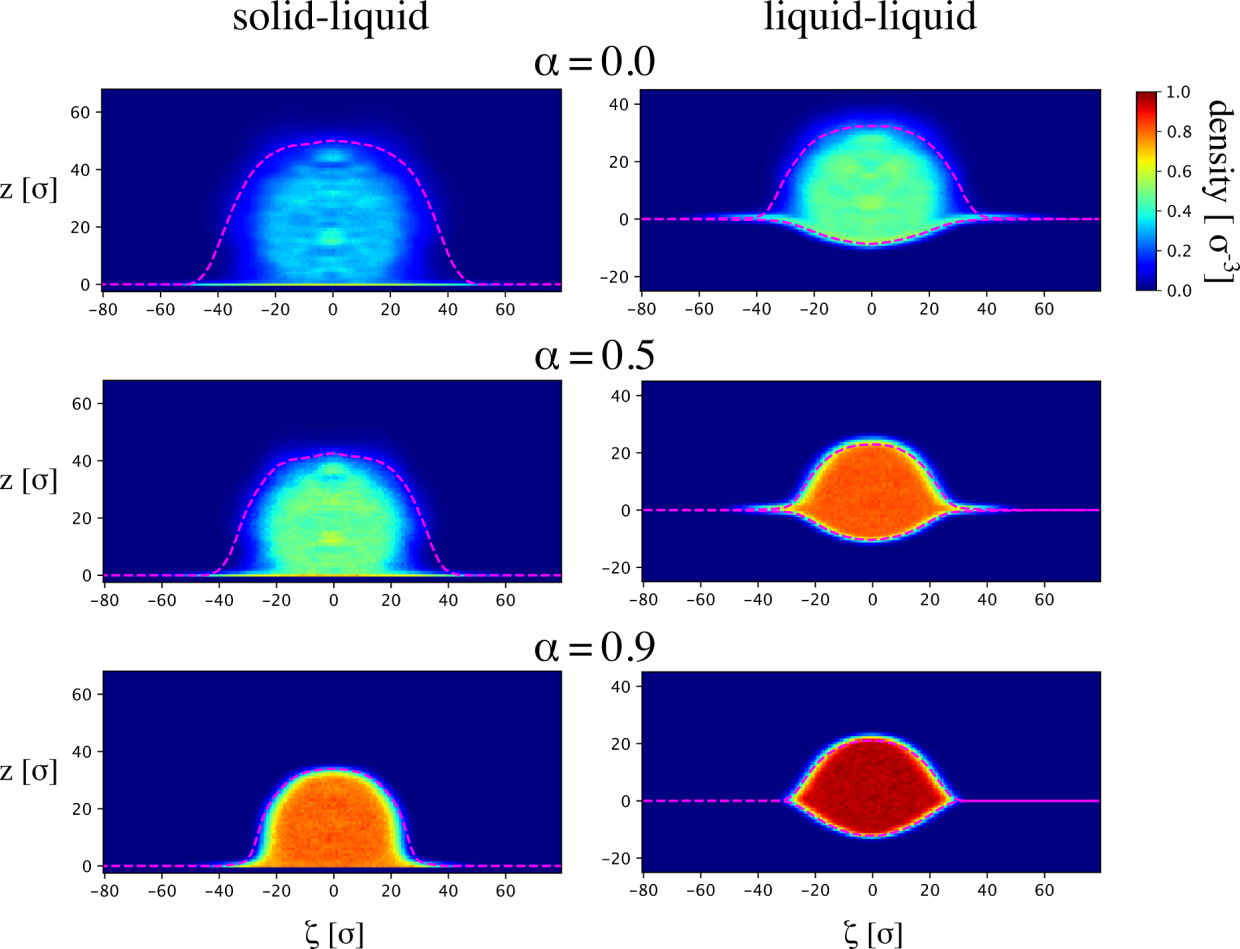
Density color-maps of a slice of a microgel at the (left) solid–liquid and (right) liquid–liquid interfaces for an increasing effective temperature α (from top to bottom), as from the color bar reported on the top right. Positive values of *z* are for the preferred water phase while negative ones are for the oil side. The dashed pink lines correspond to the height profile reported on Figure 3b.

We now move to calculate the microgel density profiles perpendicular ρ­(ζ) and parallel ρ­(*z*) to the interfacial plane, as shown in Figure 5a,b, respectively. We recall that unlike height profiles, these profiles fulfill mass conservation. Inspecting the water side from Figure 5a, we see that independently of the interface, the peak of the density profiles grows and gets narrower as the effective temperature increases, indicating the accumulation of material toward the center of the particle. The ratio between the growth in height and the reduced size at the level of the interface is such that the overall material content decreases with temperature from the preferred phase, causing an effective migration of material toward the oil side. The percentages of polymer content on the water side, also reported in the Figure 5a, are found to be 74%, 67%, and 65% for the effective temperatures α = 0.0, 0.5, and 0.9, respectively. These results align with the previously mentioned hypothesis, explaining the larger peak of the experimental and simulated height profiles *h*_*z*_(ζ) on the oil side (Figure 3). As the temperature increases, the polymer chains immersed in water collapse into the microgel core near the interface, with a small fraction overflowing into the oil side. Furthermore, the shift of material toward the oil side causes the shape of the profile in water for solid–liquid and liquid–liquid cases to diverge further with increasing temperature, as required by mass conservation. This contrasts with the trend observed when comparing the water side of the height profiles, which instead become more similar above the VPTT. These results clearly underscore that the amount of information that can be inferred from the characterization of the microgel through height profiles is quite limited with respect to the actual distribution of material within a microgel.

**Figure 5:**
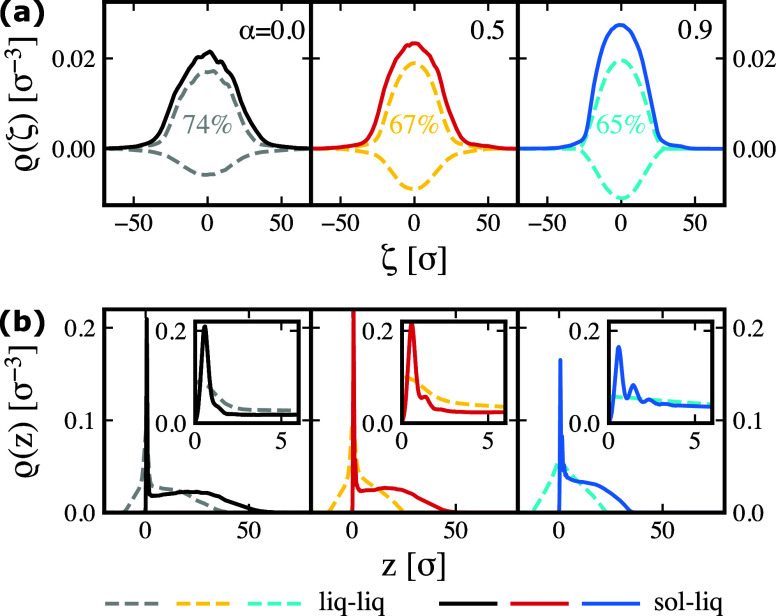
(a) Perpendicular ρ­(ζ) and (b) parallel ρ­(*z*) density profiles for liquid–liquid (dashed lines) and solid–liquid (full lines) interfaces. From left to right, the effective temperatures are α = 0.0, 0.5, and 0.9. In (a), the percentage of polymer on the water side for the liquid–liquid interface is indicated. Insets in (b) are enlargements close to the interfacial plane *z* = 0.

By looking at the density profiles parallel to the interfacial plane, ρ­(*z*), reported in Figure 5b, we clearly detect a difference regarding the polymer content close to the interface, i.e., *z* ≈ 0. While after the maximum value at *z* ≈ 0, ρ­(*z*) decreases monotonically with *z* for the microgel at the liquid–liquid interface, this is not always the case at the solid–liquid interface, where we detect the appearance of small peaks, made more evident in the respective insets. The peaks correspond to the formation of polymer layers parallel to the interface. The first layer forms due to the surface–monomer attraction, recruiting microgel material at the interface; then, as the temperature increases, the monomer–monomer attraction strengthens, further promoting the aggregation of polymer chains near the interface and leading to the formation of additional layers. Given the important role of the surface–monomer interaction for the formation of the first layer, on which the subsequent layers are formed, the presence of the peaks becomes more evident when the microgel is deposited on a more hydrophobic surface (see Section S6). However, it must be noticed that the length-scale at which the layer formation can be detected is small, being less than 4σ for the condition examined here. A closing remark on the profiles is that the temperature dependence of the height profile appears to be influenced by the cross-linker concentration. In fact, for a low-cross-linked microgel (*c* = 1.5%), we find that the height of the peak is indeed the highest for the largest studied temperature. This is shown in the Supporting Information in Section S7.

Finally, to further investigate the differences that arise between the two kinds of interfaces, we inquire the original position of the polymer chains that are mostly adsorbed. To do so, we depict in the top panels of Figure 6 the simulated microgels at the liquid–liquid and solid–liquid interface, respectively, coloring the beads at the interfaces in purple while leaving the rest in gray. Then, in the smaller panels at the bottom, we show the microgel in bulk from three different perspectives, keeping the same beads colored as those used at the interface. At the liquid–liquid interface, adsorbed beads mainly come from the central part of the microgel. Most importantly, as it is a full slice, it contains polymer chains from the shell but also from an inner region of the microgel, including some belonging to the core. Instead, at the solid–liquid interface, beads come from the bottom of the shell, far away from the center of mass. The difference in the polymer chains adsorbed at each interface suggests the possibility of interfacial tunability and selectivity, for example, by resorting to more complex microgel topologies such as composite microgels.^63–65^

**Figure 6:**
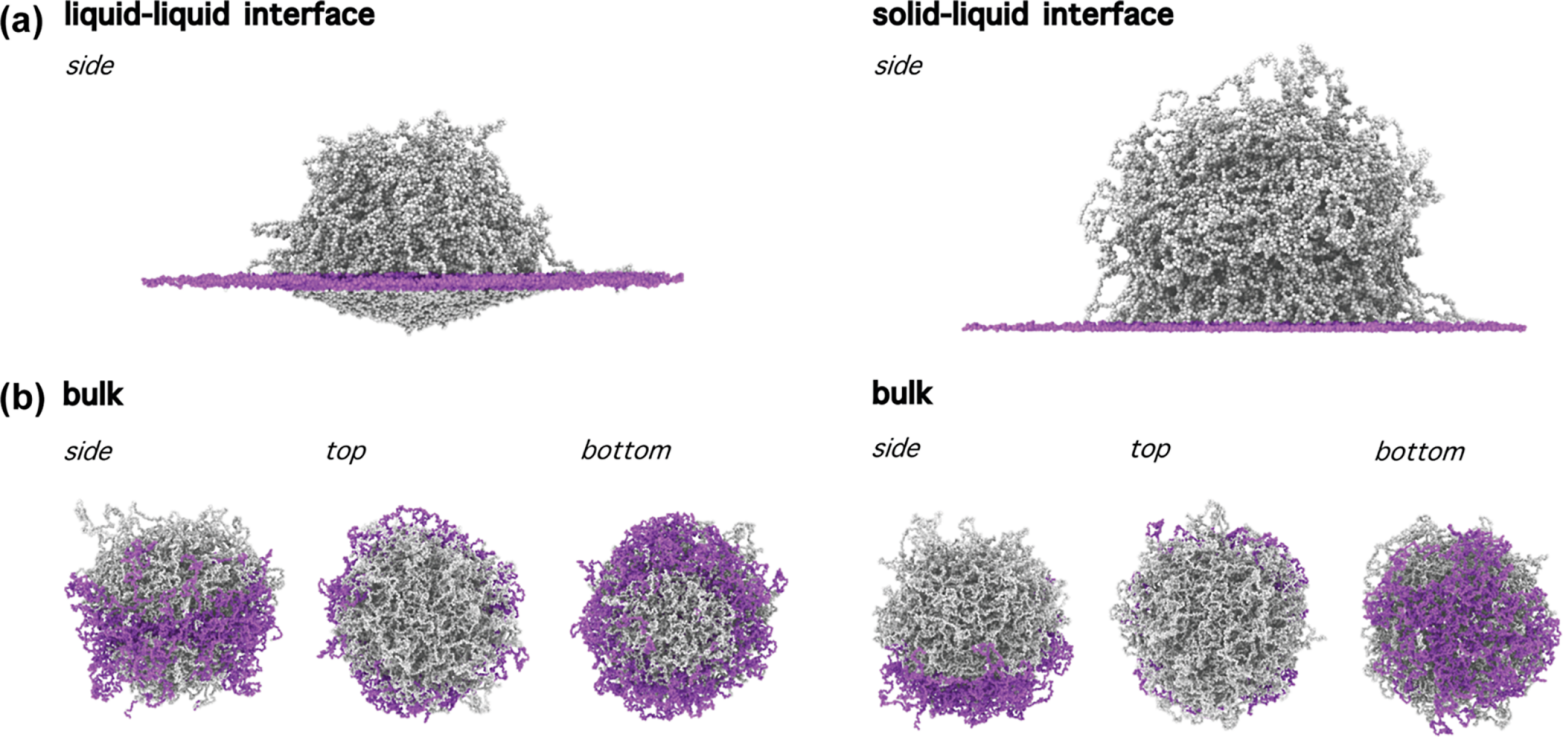
(a) Representative side-view simulation snapshots of a microgel at the (left) liquid–liquid and (right) solid–liquid interface. The microgel’s beads at the interfacial plane are colored in purple. (b) Simulation snapshots of the same microgels as in (a) but in bulk conditions, from different perspectives. The colored beads are the same as in (a).

## Conclusions

This study provides a comprehensive investigation of the structural behavior of microgels at liquid–liquid and solid–liquid interfaces, integrating in situ AFM experiments with numerical simulations. The present findings highlight fundamental differences in microgel adsorption and deformation across these two environments, shedding light on the interplay among interfacial forces, polymer–solvent interactions, and temperature effects. Understanding these behaviors is crucial for optimizing the design and functionality of microgels in interfacial applications and for determining the most relevant techniques for a thorough characterization of their structure under such conditions.

Both experimental and numerical results reveal that microgels at both interfaces exhibit a decrease in the height profiles on the water side and a reduction in lateral extension as the temperature increases. This response is consistent with the collapse of the polymer network due to worsening solvent conditions, leading to a more compact conformation. Interestingly, at liquid–liquid interfaces, a subtle increase in height is observed on the oil side with rising temperature, suggesting a redistribution of polymer chains toward the less favorable phase. Despite these variations, the height profiles in water at both interfaces become increasingly similar at higher temperatures, indicating a converging response in terms of microgel deformation.

On the contrary, density profiles reveal significant differences in the internal polymer distribution at the two interfaces. Simulations show that microgels at liquid–liquid interfaces experience a temperature-induced shift of the polymer content from the water to the oil phase. Furthermore, we observe accumulation of material in the center of the particle, which is distinctly more evident when adsorption occurs on a solid. The interplay of these two factors ultimately enhances the deviations of the profiles observed in the two environments. For the solid–liquid case, microgels also form polymer layers that are aligned parallel to the substrate, a feature that is absent at liquid–liquid interfaces. Furthermore, we noticed that the polymer subsets that participate in interfacial adsorption differ. While at solid–liquid interfaces, adsorption primarily involves the outer corona, at liquid–liquid interfaces, a broader cross-section of the microgel, including particles from the core, contributes to interfacial assembly. These differences highlight the need for complementing an overall AFM characterization with microscopic insights within the interior of the microgels, e.g., density profiles, to fully capture the rearrangements of soft, deformable particles at interfaces. At the same time, these differences also suggest possible options to tune the selectivity of microgel adsorption by controlling the structural composition. For instance, introducing a secondary polymer shell could significantly modify the adsorption behavior at solid–liquid interfaces while preserving the adsorption features at liquid–liquid interfaces. Such an approach could provide a powerful means of tailoring interfacial properties for specific applications.

In summary, by integrating experimental and computational approaches, this work demonstrates that even for individual microgels, for which the overall morphology is comparable with similar out-of-plane protrusion and in-plane extension, fundamentally different internal organizations can arise, which may have profound implications for their functional properties. Future studies could explore how modifications in microgel architecture, such as composite shells or tailored cross-linking distributions, influence interfacial adsorption and responsiveness at different interfaces. In addition, it will be important to also assess the influence of the single-particle microstructure on the types of assemblies formed by the microgels at different interfaces. Such insights will be valuable for applications in emulsification, interfacial stabilization, and soft coatings where precise control over microgel behavior at interfaces is essential.

## Supplementary Material



## Notes

Details on the gravity value for solid–liquid interface simulations, the search of the DPD parameters for the *scaled* system, additional information regarding the slope and linearity of the *a*_mx_ parameters with α, as well as their transferability to a looser cross-linked microgel, details on the hydrophobic surfaces contributions to the profiles, and DLS measurements of the particle size as a function of temperature (PDF)
