# Supplementary material for: Comparing the Structure of Microgels at Liquid–Liquid and Solid–Liquid Interfaces
Source: Langmuir. 2025 Jun 20;41(25):16674–84. doi: 10.1021/acs.langmuir.5c02599 (PMC12224307; doi:10.1021/acs.langmuir.5c02599)
Supplement: Supplementary file 1 [file la5c02599_si_001.pdf]

SI - Comparing the structure of microgels at liquid-liquid and solid-liquid interfaces

Rodrigo Rivas-Barbosa,[*,†] Fabrizio Camerin,[‡] Jacopo Vialetto,[*,¶,§] Shivaprakash N. Ramakrishna,[‖] Lucio Isa,[‖] and Emanuela Zaccarelli[*,⊥,#]

†*School of Physics and Astronomy, University of Edinburgh, EH9 3FD Edinburgh, United Kingdom*

‡*Division of Physical Chemistry, Department of Chemistry, Lund University, P. O. Box 124, SE-22100 Lund, Sweden*

¶*Department of Chemistry, University of Florence, via della Lastruccia 3, Sesto Fiorentino, I-50019 Firenze, Italy*

§*Consorzio interuniversitario per lo sviluppo dei Sistemi a Grande Interfase (CSGI), via della Lastruccia 3, 50019 Sesto Fiorentino (FI), Italy*

‖*Laboratory for Soft Materials and Interfaces, Department of Materials, ETH Zürich, Vladimir-Prelog-Weg 5, 8093 Zürich, Switzerland*

⊥*CNR Institute of Complex Systems, Uos Sapienza, Piazzale Aldo Moro 2, 00185, Roma, Italy*

#*Department of Physics, Sapienza University of Rome, Piazzale Aldo Moro 2, 00185 Rome, Italy*

E-mail: rodrigo.rivasbarbosa@ed.ac.uk; jacopo.vialetto@unifi.it; emanuela.zaccarelli@cnr.it

S1 - Gravity on Solid-Liquid Interface Simulations

In a previous work,[1] involving super-resolution microscopy and molecular dynamics simulations, an interesting fact emerged: the structure of a microgel near a hydrophilic surface is imperceptibly perturbed as compared with its state in bulk conditions. On the numerical side of that study, to maintain the microgel near a hydrophilic surface, permanent bonds between monomers and wall particles were needed. In this work, we proposed the use of a gravity-like force on the monomers to yield a similar outcome in a more realistic manner, since it is suspected that gravity plays a role in the deposition. The value of the gravity acting on the monomers is systematically sought. First, several values were tested, aiming to find one that would keep the microgel near a hydrophilic surface without perturbing it significantly relative to the bulk case. To promptly identify an approximate value, initial tests were conducted for a small $N \sim 5000$ microgel (cross-linker $c = 5\%$) for $\mathrm{F}_g = 0.0001, 0.0003, 0.0005, 0.0007, 0.001, 0.002, 0.003, 0.005$ and 0.01, *i.e.*, exploring over three orders of magnitude. The density profiles are shown in Figure S1. In this case, the profiles plotted are the (a) radial 3D $\rho^{3\mathrm{D}}(r)$, (b) its 2D projection into the interface plane $\rho^{2\mathrm{D}}(r)$, and (c) the parallel to the surface $\rho(z)$ density profiles. Interestingly, when comparing the 3D

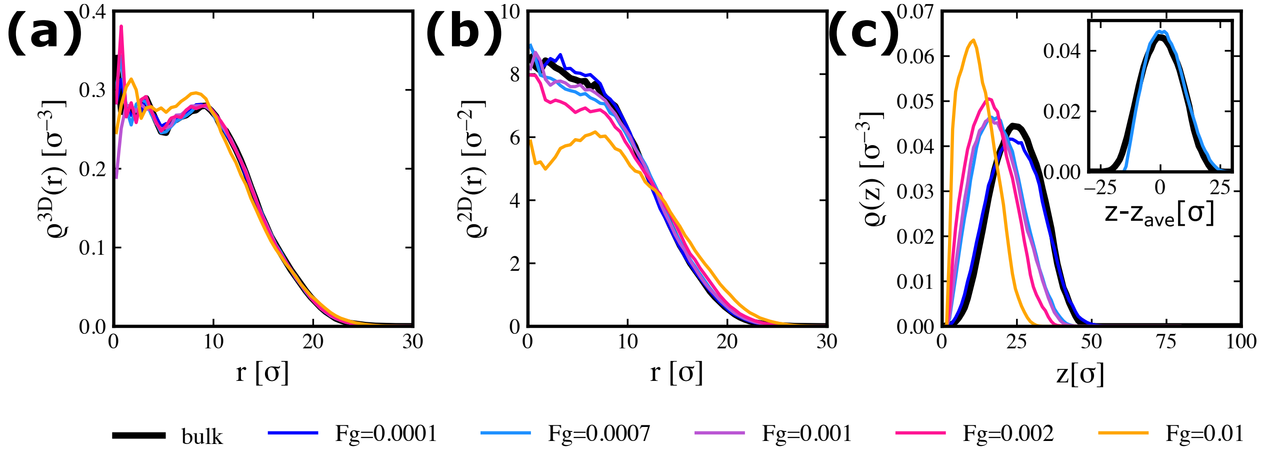

Figure S1: Density profiles for a $N \sim 5000$ microgel cross-linked $c = 5\%$ near a solid hydrophilic surface. (a) 3D $\rho^{3\mathrm{D}}(r)$, (b) 2D plane-projected $\rho^{2\mathrm{D}}(r)$, and (c) $\rho(z)$ density profile. Inset in (c) contains the bulk and $\mathrm{F}_g = 0.0007$ profiles as function of $z - z_{\mathrm{ave}}$.

density profiles $\rho^{3\mathrm{D}}(r)$ centered at the microgels center of mass (CM), no difference but for

the largest gravity value $F_g = 0.01$, having a slightly denser core, can be seen. Instead, the 2D plane projected profiles $\rho^{2D}(r)$ present more marked differences; the larger tail extension with respect to the bulk leads to discriminate $F_g > 0.001$ values. Finally, looking at the $\rho(z)$, we have evidence that even the $F_g = 0.001$ case is perceptively deformed. Incidentally, the $F_g = 0.0001$ profile diverts from the bulk, it is broader and snaphots (as well as tracing the center of mass in z) show that the microgel is actually bouncing on and off the wall. From these results, the use an external force of $F_g = 0.0007$ is found to be the preferable choice: it ensures keeping the microgel near the surface at all times but still keeping similar bulk-like properties. This is shown in the inset of Figure S1(c) where we superimposed the $\rho(z)$ profile for bulk and $F_g = 0.007$ simulations as a function of the difference between z and the average z ($z - z_{\text{ave}}$).

Next, simulations with the $F_g = 0.0007$ value for the larger $N \sim 42000$ microgel were performed with the profiles presented in Figure S2. Again an outstanding similarity in the $\rho^{3D}(r)$ can be seen: the small peak just below $10 \cdot \sigma$ is also present in the simulation with the gravity force. Small differences arise in $\rho^{2D}(r)$ and $\rho(z)$, nonetheless the main features like the extension and the height prevail. The satisfactory agreement supports again the use

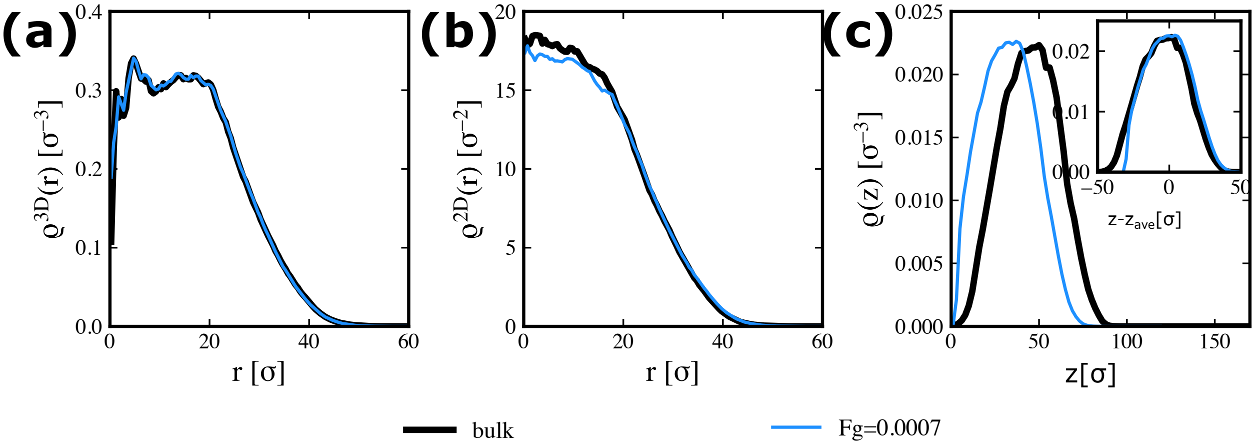

Figure S2: Density profiles for a $N \sim 42000$ microgel cross-linked $c = 5\%$ near a solid hydrophilic surface. (a) 3D $\rho^{3D}(r)$, (b) 2D plane-projected $\rho^{2D}(r)$, and (c) $\rho(z)$ density profile. Inset in (c) has the bulk and $F_g = 0.0007$ profiles as function of $z - z_{\text{ave}}$.

of the $F_g = 0.0007$ value for this larger microgel.

S2 - DPD Parameters for the Original Scaled System

To make the search faster, the a_{mx} parameters that yield an equivalent microgel configuration to that with the A approach were first sought with a smaller *scaled* system. The system contains a $N \sim 5000$ monomer microgel on a box of size $120\sigma \times 120\sigma \times 60\sigma$. The reduced density and the intra- and inter-species solvent parameters were kept the same. Snapshots of the microgel conformations at various α values are shown in the upper panels of Figure S3; starting with $\alpha = 0.0$ in the larger panel on the left, and then moving to the right by increasing α.

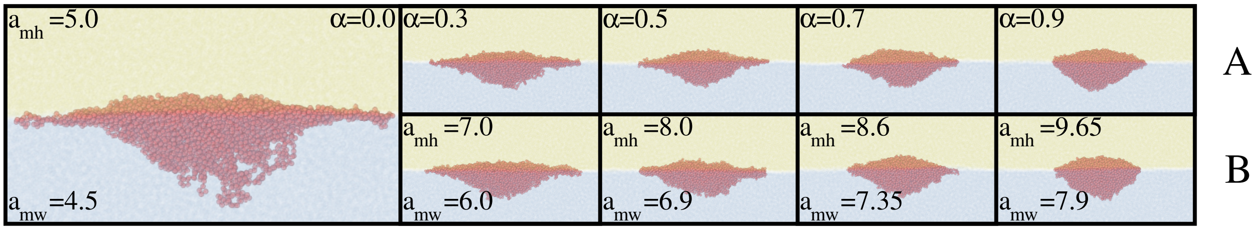

Figure S3: Snapshots of a $N \sim 5000$ microgel cross-linked $c = 5\%$ (*scaled* system) at different *temperatures* seen from the side, z positive values point down. The top (bottom) panels are snapshots when changing temperature with α (the a_{mx} DPD parameters).

The search of the a_{mx} DPD parameters was conducted with first a rough inspection between the A and B configurations, followed by a fine-tuning of the DPD parameters in order to get similar $\rho(z)$ density profiles. The lower panels of Figure S3 show the corresponding B snapshots found when attempting to get a similar conformation as that achieved using the A method. Figure S4 contains the density profiles of microgels simulated using A in symbols, and the profiles when using the a_{mx} B parameters in lines. Due to the followed strategy, in Figure S4(a) we find an excellent agreement between the α and the a_{mx} profiles. The profiles peak height as well as the extension on the oil and water side all coincide. Similarly, for the $\rho(\zeta)$ and $h_z(\zeta)$ showed in panel (b) and (c) respectively, there is as well a very good agreement between the corresponding temperature curves.

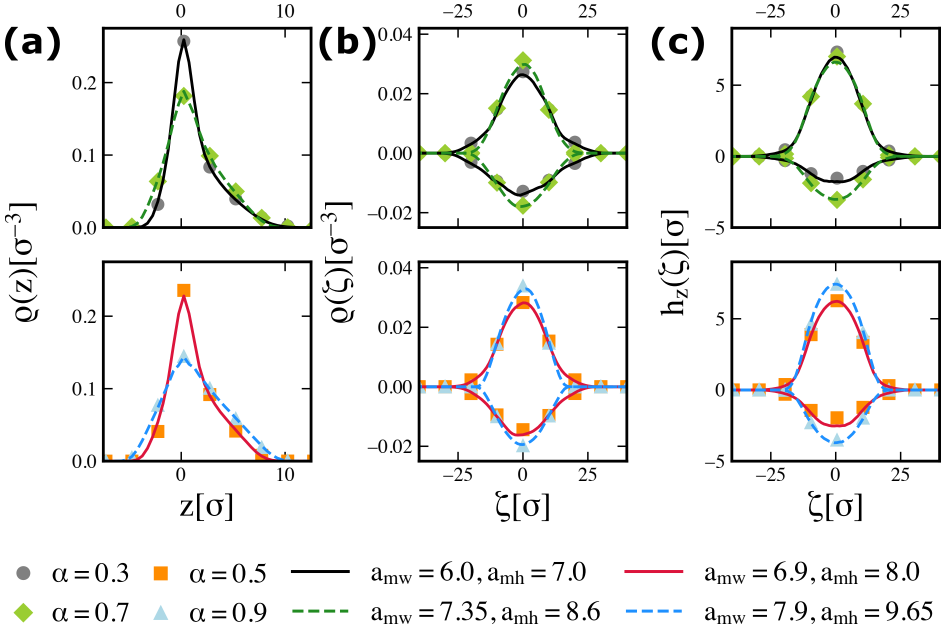

Figure S4: Profiles comparison for the *scaled* system; α in symbols, $a_{\rm mx}$ in lines. Density profile (a) $\rho(z)$ parallel and (b) $\rho(\zeta)$ perpendicular to the interface, and (c) $h_z(\zeta)$ height profile. The water (oil) side is on the positive (negative) values.

S3 - Linearity of the $a_{\rm mx}$ Parameters

The linearity of $a_{\rm mx}$ with α was examined using the smaller scaled system. To do so, the profiles of a simulation with the A method at $\alpha = 0.2$ with those of a B simulation at the estimated values of $a_{\rm mw} = 5.47$ and $a_{\rm mh} = 6.28$ were compared. The profiles are shown in figure S5. The excellent agreement between both approaches indicates the ease of predicting the $a_{\rm mx}$ parameters from their correspondent α counterpart. It is worth mentioning that the targeted temperature lays in between and very near to data points used for the fit. Furthermore, at $\alpha = 0.2$, for both monomer-solvents parameters, the data trend line and the linear fit coincide. The estimation of the $a_{\rm mx}$ may be more sensitive for larger α values.

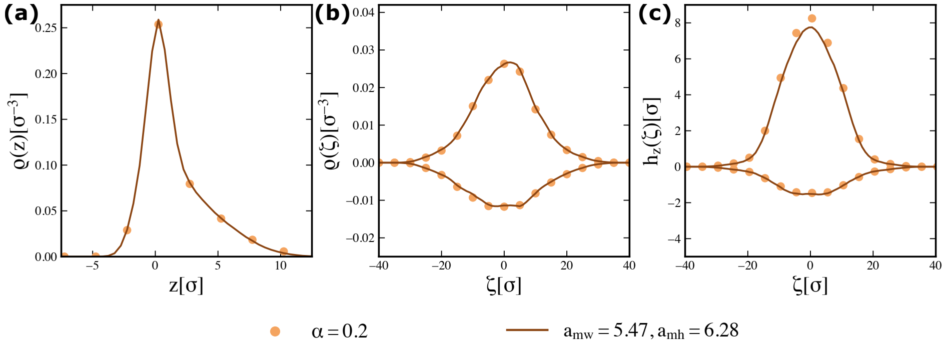

Figure S5: Profiles comparison for the *scaled* system; $\alpha = 0.2$ in triangles vs $a_{\text{mw}} = 5.47$ and $a_{\text{mh}} = 6.28$ in lines. Density profile (a) $\rho(z)$ parallel and (b) $\rho(\zeta)$ perpendicular to the interface, and (c) $h_z(\zeta)$ height profile. The water (oil) side is on the positive (negative) values.

S4 - Applicability of the a_{mx} Parameters

The transferability of the a_{mx} was also investigated for a microgel with a considerably lower cross-linker concentration $c = 1.5\%$. The snapshots of the shapes adopted by this looser microgel are shown in figure S6, it is evident a larger extension over the interface due to the larger elasticity[2] given the smaller amount of cross-linking. Still, an expected small preference to the water side can be seen. In this case, even at low α values, a fried-egg is

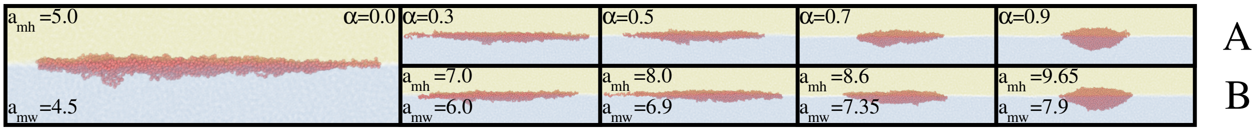

Figure S6: Snapshots of a $N \sim 5000$ microgel cross-linked $c = 1.5\%$ at different *temperatures* seen from the side, z positive values point down. The top (bottom) panels are snapshots when changing temperature with α (the a_{mx} DPD parameters).

not seen; instead, the adopted shape is flat.

The profiles comparison between both methods are shown in figure S7. The $\rho(z)$ match remarkably well but for the $\alpha = 0.7$ temperature with a few differences on the height of the peak and the water side content. The small difference at $\alpha = 0.7$ is also seen in the $\rho(\zeta)$ and $h_z(\zeta)$, particularly the a_{mx} underestimating on the water side. These discrepancies might be attributed to the fact that looser microgels with a lower percentage of cross-linkers may be more sensitive to the a_{mx} parameters. Perhaps a finer-tuning of the monomer-solvent

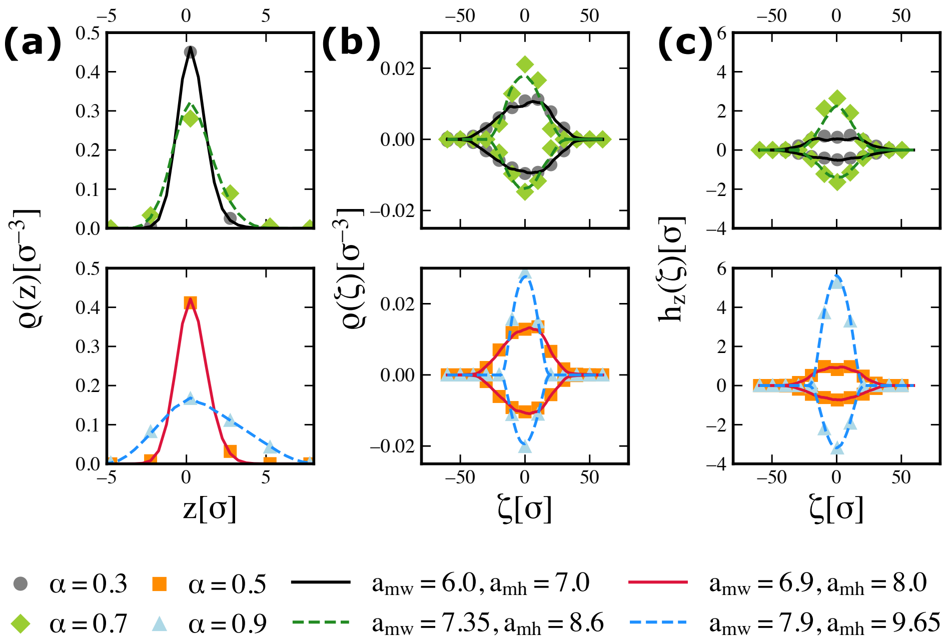

Figure S7: Profiles comparison for a $N \sim 5000$ microgel loosely cross-linked $c = 1.5\%$; α in symbols, a_{mx} in lines. Density profile (a) $\rho(z)$ parallel and (b) $\rho(\zeta)$ perpendicular to the interface, and (c) $h_z(\zeta)$ height profile. The water (oil) side is on the positive (negative) values.

interactions would significantly improve the comparison in this low cross-linked microgel without causing noticeable changes in the $c = 5\%$ microgel used for tuning. The fact that the profiles for the highest *temperature* coincide ($\alpha = 0.9$, $a_{\mathrm{mw}} = 7.9$ and $a_{\mathrm{mh}} = 9.65$) appears to support this idea, discarding the possibility of divergence in the a_{mx} parameters with temperature. In general, a good agreement is found using the same values found for the microgel with higher cross-linker concentration.

S5 - Dynamic Light Scattering (DLS) Measurements

In Figure S8 we report the hydrodynamic size (D_h) as a function of temperature, mesured by DLS, for a dilute (0.01 wt%) microgel suspension in MilliQ water at pH=6. The data confirm that the VPT occurs at a temperature $\approx 32°$ C, indicating that the influence of charged groups is negligible for the studied conditions.

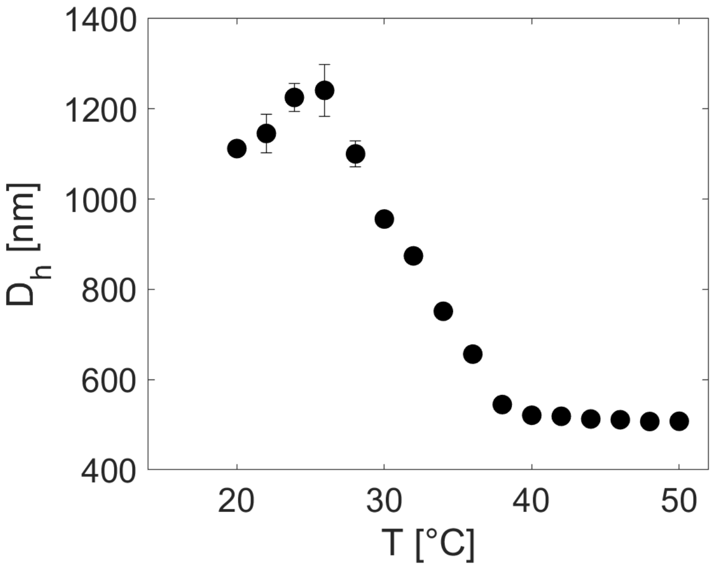

Figure S8: Swelling curve as a function of temperature for a microgel suspension in MilliQ water.

S6 - Numerical Density Profiles of Microgels near Hydrophobic Surfaces

The density profiles and the height profiles, calculated from the monomers with the maximum position away from the surface, are shown in Figure S9. The top panels contain the results for the $\alpha_{\mathrm{ms}} = 0.7$ surface, the bottom for the more hydrophobic case $\alpha_{\mathrm{ms}} = 1.0$ presented in the main text. When increasing α_{mm}, i.e., the implicit temperature driving the microgel collapse, the $\rho(z)$'s peak shrinks which implies a decrease of the surface coverage by the first monomer layer. This effect can also be seen from the slimmer $\rho(\zeta)$ and $h_z(\zeta)$ profiles; naturally, the decrease on the surface extension is smaller for the larger $\alpha_{\mathrm{ms}} = 1.0$ surface. Despite the decrease in coverage at the first layer, the microgel collapse comes with an increase of material beyond the first layer; from just after the first peak to medium z values the profiles get larger with temperature. Obviously, the microgels shrinking means a shorter extension in the $\rho(z)$ profile. Going back to the $\rho(z)$ peaks, the insets help to visualize how the first decrease with temperature is minor; instead, for $\alpha_{\mathrm{mm}} = 0.9$, the peak reduces substantially and the formation of a second and third peak can be seen. The distance between peaks, thus the

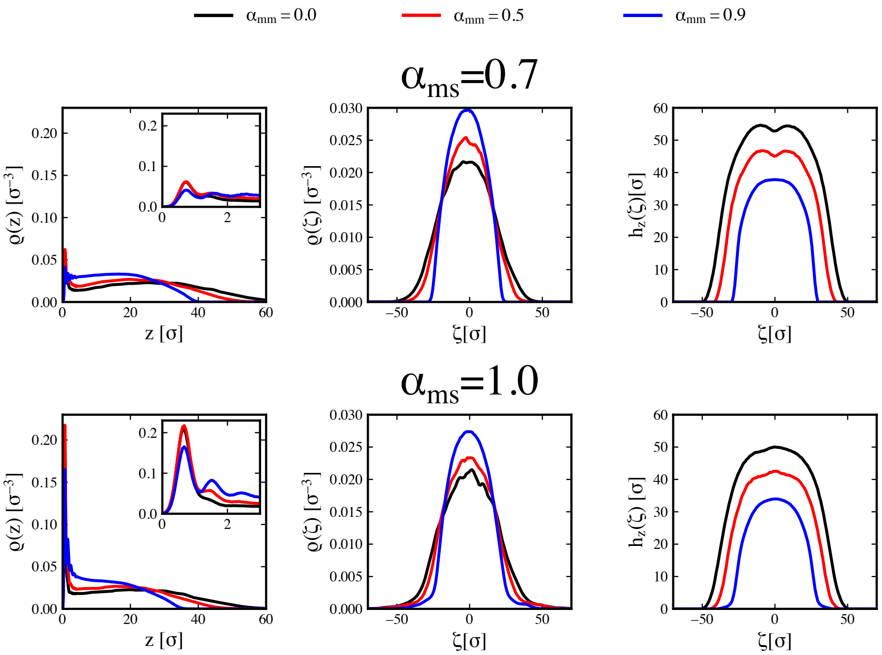

Figure S9: Profiles as function of α near a hydrophobic surface. Density profile $\rho(z)$ parallel and $\rho(\zeta)$ perpendicular to the interface, and $h_z(\zeta)$ height profile for a $\alpha_{\mathrm{ms}} = 0.7$ and 1.0 surface, respectively.

distance between monomer layers parallel to the surface, is about $0.9 \cdot \sigma$. The $\rho(\zeta)$ profiles get thinner and higher with temperature; the collapse drags material from the less cross-linked shell into the centered core. Nonetheless, the height profiles $h_z(\zeta)$ remain wider and higher when temperature is low. Although the temperature behavior is shared along both surfaces, an interesting additional feature is present for the larger $\alpha_{\mathrm{ms}} = 1.0$ surface: the $h_z(\zeta)$ profile includes some additional lateral extension that persist even for the shrunken microgel. The lateral extension is originated from the monomers attached to the surface (not permanently bonded) far away from the collapsed body. The existence of monomers far away from the xy CM is in agreement with the experimental results from a previous work;[1] it is worth mentioning again that no permanent bonds between microgel and wall are used in this study.

S7 - Behavior as a Function of Microgel Size and Crosslinker Concentration

In this section, we numerically study the behavior of the microgel at both interfaces as function of its size and crosslinker concentration. We begin first by exploring the case of a smaller microgel ($N \sim 5$k) with the same crosslinker concentration ($c = 5\%$) as in the main text. Then, we move to examiner a similarly small microgel with a lower crosslinker concentration of $c = 1.5\%$, which is much softer than the previous case.

S7a- Small Microgel $N \sim 5$k with $c = 5\%$ Crosslinker Concentration

Figure S10 shows the profiles comparison for the microgel with $c = 5\%$ crosslinker concentration at solid-liquid and liquid-liquid interfaces. Overall, the observations made for the $N \sim 42$k microgel described in the main text also apply here. Perhaps, the only noticeable differences with respect to the larger microgel are the sharper peaks on the water side but flatter on the oil side for the $h_z(\zeta)$ height and $\rho(\zeta)$ perpendicular density profiles, particularly for low temperatures $\alpha = 0.0$ and 0.5.

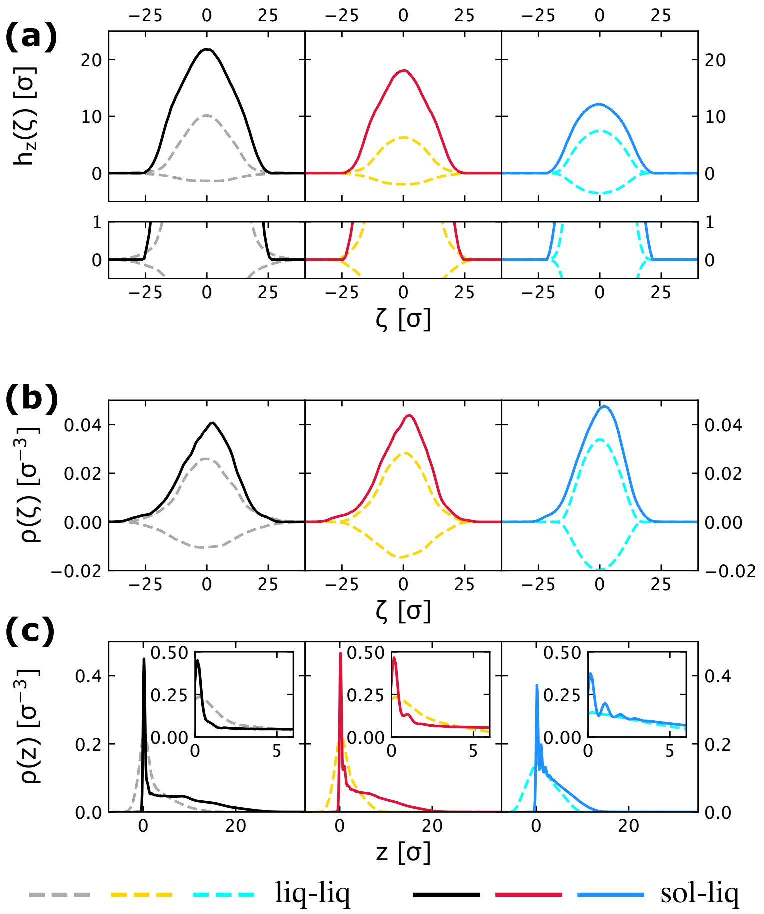

Figure S10: Profiles for a simulated microgel of $N \sim 5$k beads and crosslinker $c = 5\%$. (a) Height $h_z(\zeta)$, (b) perpendicular $\rho(\zeta)$ and (c) parallel $\rho(z)$ density profiles for liquid-liquid (dashed lines) and solid-liquid (full lines) interfaces. From left to right, the effective temperatures are $\alpha = 0.0$, 0.5, and 0.9.

S7b- Small Microgel $N \sim 5$k with Lower $c = 1.5\%$ Crosslinker Concentration

Figure S11 presents the profiles for a small microgel ($N \sim 5$k) with $c = 1.5\%$ crosslinker concentration, showing a different behavior in the the height profiles $h_z(\zeta)$ with respect to the $c = 5\%$ case. From panel (a), we can immediately see that at low temperatures the low crosslinker density plays an important role causing the height profiles not to have centered single peak, this due to the more irregular material distribution of the microgel. For the solid-liquid interface, we see a non-monotonic response of the peak with temperature, first noticing the centering of the peak and its decrease from $\alpha = 0.0$ to 0.5, only to then reach

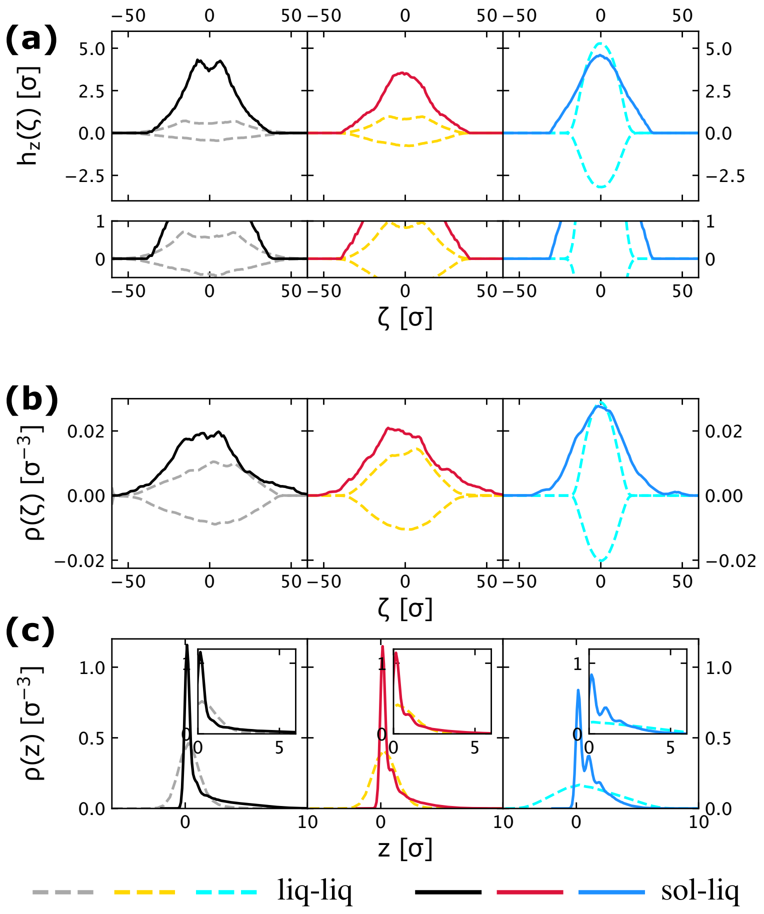

Figure S11: Profiles for a simulated microgel of $N \sim 5$k beads and crosslinker $c = 1.5\%$. (a) Height $h_z(\zeta)$, (b) perpendicular $\rho(\zeta)$ and (c) parallel $\rho(z)$ density profiles for liquid-liquid (dashed lines) and solid-liquid (full lines) interfaces. From left to right, the effective temperatures are $\alpha = 0.0$, 0.5, and 0.9.

its highest height at $\alpha = 0.9$. The decrease from $\alpha = 0.0$ to 0.5 can be explained by the compaction of the loose dangling ends with the loss of solubility. Then, if we keep rising the temperature, material from all around the microgel and not only the dangling ends gathers up near the microgels center, building up the peak observed at $\alpha = 0.9$. Passing now to the liquid-liquid interface, we observe a striking difference in the response to temperature with respect to the $c = 5\%$ microgel discussed in the manuscript. Here, the profile peak(s) on the water side increases with temperature, and in fact significantly from $\alpha = 0.5$ to 0.9. At this temperature, the attraction between microgel beads is such that the microgel compaction becomes energetically more favorable than the spread configuration that decreases the surface

tension between the two liquids.

The more irregular nature of this microgel is again captured by the perpendicular density profile $\rho(\zeta)$ in (b) where exclusively for case of the highest temperature and liquid-liquid interface we observe a more or less symmetric distribution on the ζ axis. The response with temperature for this and the perpendicular is analogous to the $c = 5\%$ case, with an increase around the center at the expense of the edges. Similarly, the parallel density profile $\rho(z)$, shown in panels (c), share the same features with the microgel with $c = 5\%$ cross-link, again emphasizing the appearance of layers for the solid-liquid interface.

References

(1) Shaulli, X.; Rivas-Barbosa, R.; Bergman, M. J.; Zhang, C.; Gnan, N.; Scheffold, F.; Zaccarelli, E. Probing Temperature Responsivity of Microgels and Its Interplay with a Solid Surface by Super-Resolution Microscopy and Numerical Simulations. *ACS Nano* **2023**, *17*, 2067–2078, PMID: 36656959.

(2) Scotti, A.; Schulte, M. F.; Lopez, C. G.; Crassous, J. J.; Bochenek, S.; Richtering, W. How Softness Matters in Soft Nanogels and Nanogel Assemblies. *Chemical Reviews* **2022**, *122*, 11675–11700, PMID: 35671377.
